# Effect of lithium administration on brain activity under an emotion regulation paradigm in healthy participants: a functional magnetic resonance imaging study

**DOI:** 10.1007/s00213-023-06395-7

**Published:** 2023-06-20

**Authors:** Pilar Artiach Hortelano, Marieke A. G. Martens, Abigail Pringle, Catherine J Harmer

**Affiliations:** 1 Department of Psychiatry, University of Oxford, Oxford, UK; 2 Oxford Health NHS Foundation Trust, Oxford, UK; 3 Wellcome Centre for Integrative Neuroimaging, University of Oxford, Oxford, OX3 7JX UK

**Keywords:** Lithium, Emotional regulation, Task fMRI, Emotional processing

## Abstract

**Rationale:**

Emotion regulation (ER) difficulties have been previously described in bipolar disorder (BD). Whilst lithium has been shown to be effective in the treatment of BD, the mechanisms underlying lithium’s effect on mood stabilisation remain unclear.

**Objectives:**

Unravelling lithium’s effect on psychological processes impaired in BD, such as ER, could address this translational gap and inform the development of new treatments.

**Methods:**

This study investigated the neural effects of lithium (800mg) on ER in 33 healthy volunteers in a double-blind between-groups design, randomised to lithium (*n*=17) or placebo (*n*=16) for 11 days. At treatment completion, participants underwent 3-Tesla fMRI scan whilst performing an ER task.

**Results:**

Reappraisal reduced negative affect across groups and led to the expected increase in frontal brain activity. Participants receiving lithium showed (1) decreased activation in prefrontal and posterior parietal cortices and connectivity between the fronto-limbic network (*Z*>2.3, *p*<0.05 corrected); and (2) increased activity in the right superior temporal gyrus (*Z*>3.1, *p*<0.05 corrected) and connectivity between the right medial temporal gyrus (MTG) and left middle frontal gyrus (*Z*>2.3, *p*<0.05 corrected) during reappraisal. Further effects of lithium were found in response to negative picture presentation, whereby an anticorrelation was found between the left amygdala and the frontal cortex, and greater connectivity between the right MTG and the bilateral medial prefrontal cortex extending into the paracingulate gyrus, compared to placebo (*Z*>2.3, *p* < 0.05 corrected).

**Conclusions:**

These results show a potential effect of lithium on ER through its effects on activity and connectivity, and further elaborate the neural underpinnings of cognitive reappraisal. Future work should investigate longer term effects of lithium on ER in BD, ultimately benefitting the development of novel and more effective treatments.

**Supplementary Information:**

The online version contains supplementary material available at 10.1007/s00213-023-06395-7.

## Introduction

Bipolar disorder (BD) is characterised by recurrent episodes of depression and mania/hypomania, affecting not only mood, but motivation, cognition, and behaviour (APA 2022). Lithium has shown to be effective in reducing the occurrence of such episodes (Licht 2012; Won and Kim 2017) in addition to having anti-suicidal, neurotrophic, and neuroprotective effects, making it the main treatment for BD since its effects were reported 60 years ago (Alda 2015). Recent efforts to understand its neurochemical properties have yielded several pharmacological pathways whereby lithium exerts its mood-stabilising effects including inositol depletion and GSK inhibition. However, the translation of these neurochemical effects to mood-stabilisation remains unclear. Understanding this translational gap is motivated by a desire to further elaborate on the underlying pathogenesis of BD; assist in the prediction of patients’ response to lithium treatment (Licht 2012); and an imperative need to develop novel and more effective treatments for the disorder, given that two out of three patients show a poor response (Hui et al. 2019), or do not respond at all (Won and Kim 2017) to mood stabilising medication.

Theoretical models of BD propose emotional regulation (ER) difficulties to contribute to its underlying psychopathology. These difficulties are proposed to lead to emotional extremes and mood dysregulation, ultimately giving rise to its characteristic pathological mood states (i.e. mania and/or depression) (Townsend and Altshuler 2012; Johnson et al. 2016; Brady et al. 2016; Li et al. 2019). ER allows the modulation of one’s response to emotional stimuli (Townsend and Altshuler 2012), with the fronto-limbic network implicated in such an effort (Dixon et al. 2017). This network comprises cortical regions including the anterior cingulate cortex (ACC), medial prefrontal cortex (mPFC), ventrolateral prefrontal cortex (vlPFC) and insula, and limbic regions including the amygdala and striatum (Townsend and Altshuler 2012; Zhang et al. 2018b). Evidence from structural and functional neuroimaging studies suggest a disruption in the fronto-limbic network may underlie ER difficulties in BD (Benedetti et al. 2011; Strakowski et al. 2012; Radaelli et al. 2015). Structurally, greater amygdala volumes (Phillips et al. 2008; Townsend et al. 2013), grey matter (GM) reductions in cortical areas such as the ACC (Malhi et al. 2013; Won and Kim 2017; Chen et al. 2018; Kato 2019), and disruption of superior white matter affecting prefrontral areas (i.e. dlPFC, vlPFC, and mPFC) and left parietal cortex (Zhang et al. 2018b) have been described in BD when compared to healthy controls. Functionally, ER difficulties in BD have been studied employing an ER paradigm that uses reappraisal as a strategy to regulate one’s emotions to disturbing stimuli. This strategy, whereby an individual consciously attempts to reinterpret the processed stimulus to reduce its emotional effect, is one of the most common and successful strategies for ER (Buhle et al. 2014). In healthy individuals, reappraisal recruits domain-general cognitive control areas, namely anterior PFC, dlPFC, vlPFC, and the dorsomedial prefrontal cortex (dmPFC), in addition to posterior parietal areas (i.e. angular gyrus (AG), supramarginal gyrus, and superior parietal lobe) involved in attentional processes (Ochsner et al. 2002; Phan et al. 2005; Buhle et al. 2014). These areas are then hypothesized to either (1) recruit the ventromedial prefrontal cortex (vmPFC) to modulate regions involved in emotional processing, such as the amygdala (Diekhof et al. 2011), or (2) engage lateral temporal regions (i.e. superior and medial temporal gyrus), associated with language processing, to alter the semantic and perceptual meaning of the stimulus, ultimately changing its emotional significance (Ochsner et al. 2012; Buhle et al. 2014). In patients with BD, reappraisal of negative stimuli is characterised by decreased bilateral activation in vlPFC, anterior and posterior cingulate cortex, and dlPFC, as well as medial frontal gyrus when compared to healthy controls (Townsend et al. 2013). Additional evidence shows weaker dlPFC-amygdala connectivity, which might reflect failure of prefrontal areas to engage in effective ER (Zhang et al. 2018a, 2020). Overall, structural deficits due to GM reductions in the ACC, together with disruption of superior white matter affecting key areas for ER, have been hypothesised to be linked to ER impairments in BD (Benedetti et al. 2011).

Unravelling lithium’s effect on core psychological processes impaired in BD, such as ER, can further elaborate on the mechanisms whereby lithium leads to mood stabilisation in the disorder. Here we used an experimental medicine approach with healthy volunteers which allowed the direct assessment of lithium’s effects unconfounded by changes in clinical symptomology. The present study therefore assessed brain activity as well as an explorative analysis of connectivity during ER in healthy participants administered either lithium or placebo for 10–12 days in a double-blind randomised design. It was hypothesised that:

1. lithium would increase activation in PFC and posterior parietal areas during active reappraisal (enhanced cognitive control) and/or decrease activation in amygdala and temporal regions (decreased emotional reactivity) and
2. lithium treatment would result in greater connectivity between critical nodes of this network, in line with the proposed effects on ER.

## Methods

### Participants

Thirty-six healthy participants (18 males, aged 18–31) were recruited through advertising. Following the local ethics committee guidelines (NRES committee South central–Oxford REC B 10/H0605/71), written and oral consent was obtained. Participants had a body mass index of 19–30, were physically fit as assessed by a medical doctor, and had normal laboratory values of thyroid and renal function. Additionally, females tested negative on a pregnancy test and were using two forms of contraception. Participants were excluded if they took any psychotropic medication, had any past or current psychiatric including mood and anxiety disorders, had any medical contra-indication (see the Supplementary Material), had current or past history of drug or alcohol dependency, smoked more than 5 cigarettes a day, had dyslexia, had any contra-indication to magnetic resonance imaging (MRI) scanning, or were left-handed. A total of 3 participants were further excluded from the experiment and/or analysis due to treatment non-compliance (1x), unexpected adverse effect (1x; reported anxiety increase), and incomplete MRI session (1x), leaving a total of 33 participants for analysis.

### Experimental design and procedure

The present study followed a double-blind randomised design (as previously reported in Volman et al. 2021). An independent qualified researcher performed the randomisation on a 1:1 ratio for treatment (placebo/lithium) and gender (male/female). Lithium (“Priadel” prolonged release tablet) or placebo were administered orally at night for 11 days (±1 day) on identical capsules. Lithium dosage was increased on a gradual fashion (day 1: 400 mg; day 2: 600 mg; days 3–11: 800 mg) following previous procedures (Kohno et al. 2007; Monkul et al. 2007) to achieve steady state during the testing sessions.

As previously described in Volman et al. (2021), participants visited the lab on three occasions. The initial assessment comprised a medical and psychiatric screening, as well as a blood drawing for testing thyroid stimulating hormone and creatine. Eligible participants were then asked to return for a baseline assessment in which the Beck Depression Inventory (BDI) (Beck et al. 1961), State-Trait Anxiety Inventory (STAI; (Spielberger 1983), Mood Disorder Questionnaire (MDQ; Hirschfeld et al. 2000), National Adult Reading Test IQ Scale (Nelson and Willison 1982), Eysenck Personality Questionnaire (Eysenck and Eysenck 1975), and Emotion Regulation Questionnaire (ERQ; Gross and John 2003) were completed. Females were additionally required to take a pregnancy test at this stage. Upon completion, participants received the full treatment (lithium/placebo), the Befindlichskeit scale (BFS; Pichot and Olivier-Martin 1974), the Positive and Negative Affect Scale (PANAS; Tran 2013), the Bond and Lader Visual Analogue Scales (Bond and Lader 1974), and side-effects questionnaires, which had to be filled each day during treatment. Participants were contacted on days 3 and 5 to ensure no treatment side effects and check appropriate compliance of the dosage regimen. Following the last treatment day, participants returned for testing. Blood was drawn to monitor lithium levels and participants underwent a behavioural and MRI session that lasted approximately 120 min. In the behavioural session, the BDI, STAI state, and MDQ as well as a battery of tasks (reported elsewhere) were completed. In the MRI session, functional and structural data were acquired whilst participants completed the ER task, the monetary incentive delay task (Volman et al. 2021), the checkerboard control task (Volman et al. 2021), and an MR spectroscopy scan (not included here).

### Tasks

An adaptation of the original ER paradigm (Phan et al. 2005), previously used by Reinecke et al. (2015), was employed. In the task, participants were presented with 8 blocks of 5 negatively valenced images in each block (mean valence rating of 2.8±1.7, mean arousal ratings of 6.0±2.2 on 9-point Likert scales from 1=unpleasant/low arousal to 9=pleasant/high arousal) to which they were instructed to alternatively maintain (naturally experience the emotional state evoked), or reappraise (downregulate the provoked negative affect) through cognitive reappraisal. Employment of cognitive reappraisal was trained prior to the scan session. Following each block, participants had to rate on a 4-point rating scale (1=neutral; 4=negative) the intensity of the negative affect experienced. A more detailed description of the task as well as an illustrative figure can be found in supplementary material (Figure S1 supplementary material). Valence, arousal ratings, and scene content matched between conditions and the order of the pictures within each condition remaining constant across all participants. The total duration of the task was of approximately 10 min.

A checkerboard control task (CCT: see Volman et al., 2021) was used to control for treatment-related possible cofounders on brain activation.

### MRI acquisition

Bold-oxygenation-level-dependent (BOLD) functional MRI (fMRI) data was acquired on a 3-Tesla MRI scanner (Magnetom, Siemens Medical systems) with a 32-channel head coil. Functional images during the ER task consisted of 45 T2-weighted echoplanar imaging (EPI) slices (TR=3000ms, TE=30ms, flip angle=90°, field of view=192 mm, voxel size=3×3×3mm, 200 volumes, acquisition time (TA)=10 min, 6 s). Functional images during the CCT consisted of 45 T2-weighted EPI slices (TR=3000 ms, TE=30 ms, flip angle=87°, field of view=192 mm, voxel size=3×3×3mm, 120 volumes, TA=6 min, 6 s). Additionally, fieldmaps volumes (magnitude and phase difference images) were acquired (echos at 5.19 and 7.65 ms, TR=488, flip angle=60°) to capture the inhomogeneities in the magnitude field. Structural scans were acquired via T1-weighted MR images (TR=2040 ms, TE=4.7 ms, flip angle=8°, field of view=192 mm, voxel size=1×1×1 isotropic, TA=5 min, 56 s).

### Analysis of fMRI data

Data were analysed using FSL (FMRIB Software Library v6.05; www.fmrib.ox.ac.uk/fsl). Structural anatomical scans were brain extracted using FSL’s Brain Extraction Tool BET (Smith 2002). Fieldmap magnitude images were brain extracted by first registering these to their high-resolution structural images, inversing the created matrix, applying such matrix to the brain extracted structural mask with FMRIB’s Linear Registration Tool (FLIRT) (Jenkinson and Smith 2001; Jenkinson et al. 2002), and applying this mask to the whole brain magnitude image. These were then used to create a fieldmap rads image with the fieldmap phase difference image using the fsl_fieldmap_prepare tool.

Pre-processing of each participant’s functional data was done with FEAT (FMRI Expert Analysis Tool), part of FSL. This included motion correction using MCFLIRT (Jenkinson et al. 2002); spatial smoothing using Gaussian kernel of full width at half maximum 5mm; grand-mean intensity normalisation of the entire 4D dataset by a single multiplicative factor; high-pass temporal filtering of the functional timeseries at 90s; fieldmap distortion correction (Jenkinson 2003, 2004); registration of the functional images to their high-resolution structural images with Boundary-Based Registration using FLIRT (Jenkinson and Smith 2001); and registration of the structural images to Montreal Neurological Institute (MNI)-152 standard space using linear registration with 12 degrees of freedom (DOFs), further refined using FNIRT non-linear registration with 10-mm resolution (Andersson et al. 2007a, b).

Regarding the ER task, lower-level analysis was carried out to observe within-subject differences in brain activity across the two conditions by including the following contrasts: (1) maintain vs. baseline (M>B) to identify brain regions active when asked to naturally experience the emotion elicited; (2) reappraise vs. baseline (R>B) to identify brain regions active during voluntary suppression of negative affect using reappraisal techniques; (3) reappraise vs. maintain (R>M) to identify brain regions with greater activation when reappraising as compared to maintain; (4) maintain vs. reappraise (M>R) to identify brain regions with greater activation whilst maintaining as compared to reappraise; and (3) overall picture blocks vs. baseline (M+R>B), to identify brain regions activated in response to negative images.

A custom 3-column format convolved with a gamma haemodynamic response function and its temporal derivative were used to model the data. Time-series statistical analysis was carried out using FILM with local autocorrelation correction (Woolrich et al. 2001). Motion traces detected by MCFLIRT were included in the model as nuisance regressors to account for motion. Differences between groups in absolute and relative motion were tested for using Mann-Whitney non-parametric analysis. Groups did not differ in absolute nor relative motion (all *U*’s>94, all *p*’s >0.130).

In the higher-level analysis for the ER task, the contrast of parameter estimates (COPEs), their variance (VARCOPEs) and DOFs from the lower-level analysis were introduced into the analysis. Using a mixed-effects analysis with FLAME1+2 across the whole brain, the following contrasts were analysed: (1) placebo>lithium (1, −1); (2) lithium>placebo (−1, 1); and (3) mean activation and deactivation across both treatment groups (1, 1; −1, −1). Due to lithium’s potential to promote GM changes, GM images of each participant were extracted using FMRIB’s Automated Segmentation tool (Zhang et al. 2001). These were then registered to standard space, smoothed to match the intrinsic smoothness of the fMRI data (2.63mm), voxel-wise demeaned across all subjects, and added to the general linear model (GLM) of the ER task to remove any potential structural differences explaining the BOLD contrast differences. Significant activations were identified using cluster-based thresholding of statistical images with a height threshold of *Z*>3.1 and a family wise error (FWE)-corrected cluster significance threshold of *p*<0.05. Clusters thresholded at *Z*>2.3 *p*<0.05 were also reported for completeness and to compare the present results with previous studies using this less stringent statistical threshold (Worsley 2001). See Volman et al. (2021) for details on CCT analysis.

Small volume correction (SVC) analysis was performed using several regions of interests (ROIs) known to be involved in emotional processing and cognitive reappraisal focused on the amygdala, vmPFC, vlPFC, and middle temporal gyrus (MTG). Bilateral amygdala’s anatomical masks were created using the probabilistic map thresholded at 50 provided by the Harvard-Oxford Structural Atlas in FSL. Spherical masks of 10-mm radius were created for the bilateral vmPFC, vlPFc, and MTG using coordinates previously reported (Buhle et al. 2014; right vmPFC: *x*=6, *y*=40, *z*=−20; left vmPFC; *x*=−6, *Y*=40, *z*=−20; right MTG: *x*=56, *y*=−32, *z*=0; left MTG: *x*=−56, *y*=−32, *z*=0; Kohn et al. 2014: right vlPFC *x*=50, *y*=30, *z*=−8; left: *x*=−42, *y*=22, *z*=−6). Significant activations were identified using cluster-based thresholding of statistical images with a height threshold of *Z*>3.1 and a FWE-corrected cluster significance threshold of *p*<0.05.

Exploratory connectivity analysis was performed to investigate a seed ROIs’ relationship with other brain areas throughout the task using psychophysiological interactions analysis in FSL (O’Reilly et al. 2012). PPI detects task-specific increases in the relationship between a seed region of interest and the rest of the brain, measured in terms of the strength of regression of activity in one region on another. However, there is no implication that the seed region is the driver rather than the driven area, or whether the connection is direct, rather than mediated by other areas (O’Reilly et al. 2012). vmPFC and MTG masks for this analysis were based on the Harvard-Oxford atlas as well. Standard masks were first transformed into individual’s standard space, then thresholded (0.5 similar to SVC task analysis) and binarized. Time-series of each mask were extracted and entered in the lower-level analysis as a regressor to identify voxels where a significant effect is explained by such regressor. Task regressors were added (maintain, reappraise and instructions), in addition to the interaction between the masks’ time-series and the task conditions (maintain × mask time-series and reappraise × mask time-series). Contrast images were introduced in the higher-level analysis to identify brain differences in connectivity across treatment groups. Significant activations were identified using cluster-based thresholding of statistical images with a height threshold of *Z*>3.1 and *Z*>2.3 and a FWE-corrected cluster significance threshold of *p*<0.05. BOLD parameter estimates of significant whole-brain or ROIs interactions were further explored plotted for visual inspection.

### Statistical analysis

Statistical analysis was performed with IBM SPSS 22 Software, with significant levels set at *p*<0.05. Between-group differences on the sample’s tendency for ER (ERQ questionnaire) were analysed with an independent samples *T*-test after testing for normality. Treatment-derived differences on self-reported ratings of negative affect experienced throughout the task were further analysed with a mixed-model ANOVA, within-subject factor condition (maintain vs. reappraise), and between-subject factor treatment (lithium vs. placebo). Post hoc analysis of the ANOVAs was carried out with pairwise comparisons.

## Results

### Demographical characteristics and questionnaire measures

Sociodemographic, clinical, and personality characteristics of participants of the final sample (*n*=33) can be found in Table 1. Both groups had higher scores in the ERQ-reappraise, indicating that reappraisal was their usual technique for ER in daily life. As previously reported in Volman et al. (2021), no significant time by treatment interactions were identified in the BDI, STAI-state, and MDQ questionnaires. Similarly, no significant group differences in the change in side effects, visual-analogue scale, PANAS, and BFS scores were found.

**Table 1:** Demographic and clinical characteristics of the sample

*Demographics*		
* N* (f/m)	16 (8/8)	17 (9/8)
Age	21.81 (3.60)	21.88 (2.67)
Years in education	15.69 (1.08)	16.00 (1.66)
*Baseline clinical measures.*		
NART	117.11 (5.08)	117.38 (3.79)
BDI	1.06 (1.88)	2.76 (4.09)
STAI-trait	29.25 (5.71)	31.65 (8.69)
STAI-state	26.94 (5.07)	31.47 (9.74)
EPQ-neuroticism	3.31 (3.09)	6.24 (4.96)
EPQ-psychoticism	2.44 (1.99)	2.12 (1.32)
EPQ-lie/social desirability	6.94 (2.96)	8.35 (2.94)
EPQ-extraversion	16.31 (3.07)	14.24 (4.68)
MDQ	1.94 (2.49)	2.59 (2.94)
ERQ-reappraise	30.80 (4.53)	30.64 (3.17)
ERQ-supress	11.93 (3.75)	15.29 (6.37)
Lithium levels	0.7 (0.2)	0 (0)

*N* sample, *(f/m)* female-to-male ratio, *SD* standard deviation, *NART* National Adult Reading Test, *BDI* Beck Depression Inventory, *STAI* State-Trait Anxiety Inventory, *EPQ* Eysenck Personality Questionnaire, *MDQ* Mood Disorder Questionnaire

### Lithium levels

Participants in the lithium group had significantly higher levels of lithium in their serum (*p* <0.001, *t*(31)=11.309; mean lithium=0.7, SD=0.2; mean placebo=0, SD=0) as reported in Volman et al. (2021). This ensures treatment compliance in this group and was within the optimal serum levels of lithium in BD maintenance treatment (Volkmann et al. 2020).

### Affective ratings

Analysis of the affect ratings showed a significant condition effect (*F*(1)=37.629, *p*<0.001, ηp2=0.565). That is, during maintain blocks (mean=2.545, SD=0.113), negative affect ratings were significantly higher than during reappraisal blocks (mean=1.849, SD=0.112; M-R difference=0.697) across treatment groups. These results show that participants successfully implemented cognitive reappraisal, reducing the negative affect elicited by negative stimuli. No significant effect of treatment nor treatment by condition interaction arose (all *F*’s>1.5, *p*’s<0.250).

### fMRI

#### Whole-brain analysis

##### Main effect of task (reappraise vs. maintain, across groups)

**Fig. 1:**
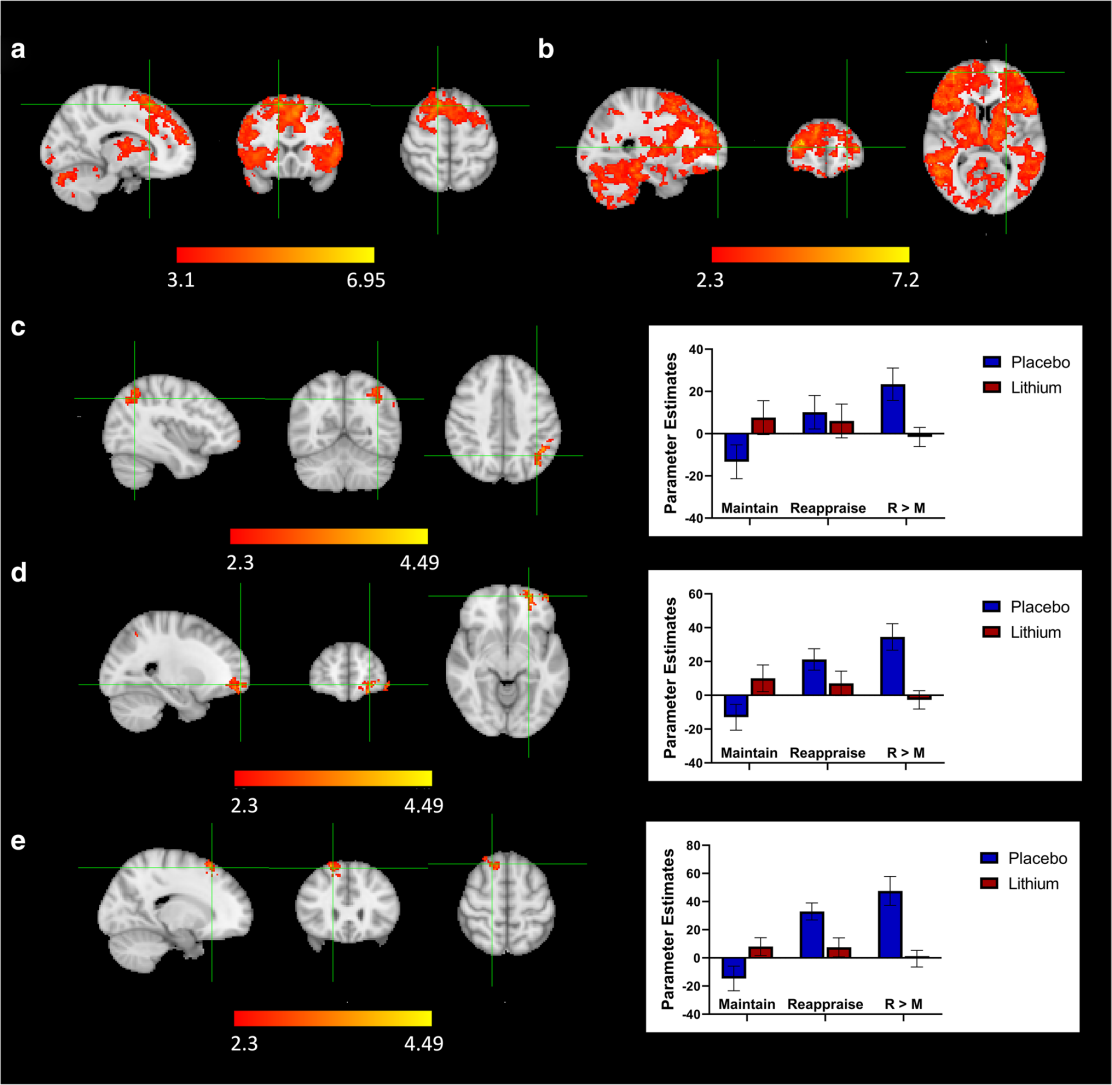
Whole-brain analysis results. **a**, **b** Main effect of task: significant increased activation in brain areas including the bilateral ACC, anterior PFC, supplementary motor cortex, dmPFC, dlPFC, vlPFC, lateral OFC, insular cortex, temporoparietal junction, superior and middle temporal gyrus, lateral occipital cortex, occipital cortex, cerebellum, thalamus, pallidum and caudate, and left supramarginal gyrus when reappraising compared to maintaining across groups. **c** Group × Task Interactions at *Z*>2.3. Significant increased activation in the left angular gyrus towards supramarginal gyrus, **d** left anterior prefrontal cortex, and **e** right superior frontal gyrus when reappraising compared to maintaining in placebo compared to lithium. In addition to the parameter estimates for the reappraise greater than maintain contrast, the parameter estimates for both maintain and supress vs baseline were also extracted for completeness. Error bars represent SEM

In order to identify which brain regions were differentially activated between conditions, maintain and reappraise conditions were compared across both groups. Significant brain activations were observed in a network of areas reported previously (Ochsner et al. 2002; Phan et al. 2005; Reinecke et al. 2015). Reappraisal increased activation in bilateral ACC, anterior PFC, supplementary motor cortex, dmPFC, dlPFC, vlPFC, lateral orbitofrontal cortex (OFC), insular cortex, temporoparietal junction, superior and middle temporal gyrus, lateral occipital cortex, occipital cortex, cerebellum, thalamus, pallidum, caudate, and left supramarginal gyrus (Table S1 supplementary material; Fig. 1 a, b). Together, these findings confirm this task engaged brain regions pertaining to a network implicated in ER.

##### Group × task interaction (reappraise vs. maintain; placebo vs. lithium)

A preliminary whole brain analysis at *Z*>2.3, *p* < 0.05 corrected, revealed reduced BOLD activation in three separate clusters: the left AG extending towards supramarginal gyrus (421 voxels, *Z*max = 3.84, MNI *x*=−46, *y*=−52, *z*=40, *p*=0.002); the left anterior PFC (303 voxels, *Z*max = 4.45, MNI *x*=−24, *y*=58, *z*=−8, *p*=0.019) and the right superior frontal gyrus (269 voxels, *Z*max = 3.89, MNI *x*=20, *y*=26, *z*=60, *p*=0.038), in those receiving lithium compared to placebo, when cognitive strategies of reappraisal were implemented compared to passively viewing the pictures (maintain condition; Fig. 1 c, d, e). There were no significant differences between the placebo and lithium group for any of the other contrasts (maintain greater than baseline, suppress greater than baseline and mean of maintain and suppress = aversive picture blocks greater than baseline).

No significant group differences were found for negative pictures > baseline (M+R>B) at the whole-brain level.

#### SVC analysis

##### Main effect of task (*Z* > 3.1, *p* < 0.05 corrected; reappraise vs. maintain; across groups)

Across groups, the reappraisal condition increased activity in bilateral amygdala and bilateral MTG extending into superior temporal gyrus (Table S2 supplementary material) compared to maintain. No significant findings in bilateral vmPFC were seen.

##### Group × task interaction (*Z* > 3.1, *p* < 0.05 corrected; reappraise vs. maintain; lithium vs. placebo)

There was a significant group **×** task interaction in the right MTG extending into superior temporal gyrus (cluster: 19 voxels, MNI *x*=52, *y*=−32, *z*=4, *Z*max =4.33, *p*=0.0058). That is, participants in the lithium group showed significant increased activation in the right superior temporal gyrus when reappraising relative to maintain, compared to placebo (Table S2 supplementary material). No significant findings in other ROIs arose.

No significant group differences were found for negative pictures > baseline (M+R>B) for any of the ROIs.

#### Connectivity analysis

##### Main effect of task (*Z* > 2.3, *p* < 0.05 corrected; reappraise vs. maintain, across groups; Fig. 2)

Activity in the right amygdala during reappraisal significantly correlated with deactivation (negative mean maintain and suppress) in the left lateral occipital cortex/superior + inferior parietal lobule extending into MTG (cluster: 1821 voxels, MNI *x*=−24, *y*=−84, *z*=30, *Z*=3.75, *p*<.001), right lateral occipital cortex/inferior parietal lobule extending into bilateral intracalcarine cortex, MTG, AG, and left precuneous cortex (cluster 1455 voxels, MNI *x*=40, *y*=−76, *z*=24, *Z*=3.99, *p*<0.001), right precentral gyrus extending into middle frontal gyrus (Brodmann area (BA) 6 + 9) (cluster: 302 voxels, MNI *x*=40, *y*=−4, *z*=46, *Z*=3.22, *p*=0.0099).

**Fig. 2:**
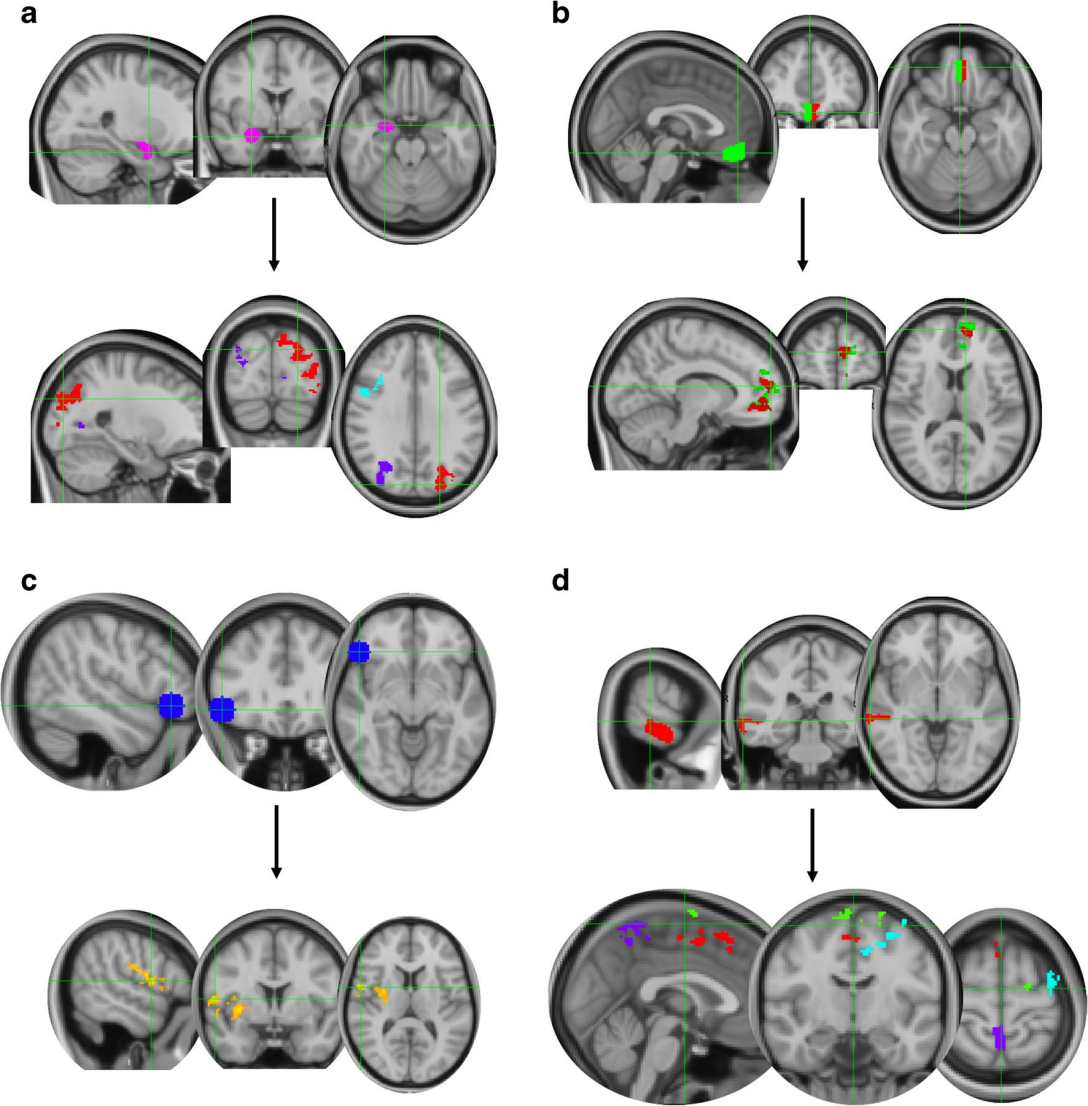
Whole-brain connectivity analysis. Main effect of task (*Z* > 2.3, *p* < 0.05 corrected). **a** Right amygdala’s activity (pink) significantly correlated with deactivation in a cluster spanning the left lateral occipital cortex (red), the right lateral occipital cortex (purple), and right precentral gyrus extending into middle frontal gyrus (BA6+9; blue) when reappraising compared to maintaining, across groups. **b** Right vmPFC (green) significantly correlated with deactivation in a cluster covering the left medial PFC to the vmPFC (green) when reappraising compared to maintaining, across groups. Left vmPFC (red) significantly correlated with deactivation in a cluster covering the left vmPFC towards the medial PFC (red) when reappraising compared to maintaining, across groups. **c** Right vlPFC’s (blue) activity significantly correlated with deactivation in the right precentral gyrus extending into central opercular cortex, insular cortex, and putamen (yellow). **d** Right MTG’s activity (red) significantly correlated with deactivation in four clusters spanning the bilateral superior frontal gyrus towards supplementary motor cortex and ACC (red), bilateral precuneous cortex extending into pre-central gyrus (purple), the left precentral gyrus towards the middle frontal gyrus, and the bilateral superior frontal gyrus (green)

In addition, activity in the left amygdala was significantly correlated with deactivation in the right lateral occipital cortex (cluster: 285 voxels, MNI *x*=34, *y*=−78, *z*=28, *Z*max=3.18, *p*=0.0213) and left lateral occipital cortex, extending into the occipital pole and superior parietal lobule (cluster: 257 voxels, MNI *x*=−24, *y*=−86, *z*=34, *Z*max=3.46, *p*=0.0389). Activity in the right vmPFC was significantly correlated with deactivation in the left medial PFC to the vmPFC and paracingulate gyrus during reappraisal (cluster: 620 voxels, MNI *x*=−16, *y*=62, *z*=12, *Z*=3.61, *p*<.001). Similarly, activity in the left vmPFC significantly correlated with deactivation of the left vmPFC towards the medial PFC and paracingulate gyrus (cluster: 316 voxels, MNI *x*=−10, *y*=58, *z*=12, *Z*=3.74, *p*=0.007) during reappraisal.

Activity in the right vlPFC was significantly coupled with deactivation in the right precentral gyrus extending into central opercular cortex, insular cortex, and putamen (785 voxels, MNI *x*=56, *y*=2, *z*=10, *Z*max=3.62, *p*<0.001).

Activity in the right MTG was significantly correlated with deactivation in four clusters spanning the bilateral superior frontal gyrus (BA6) towards the supplementary motor cortex and ACC (BA24; cluster 1: 546 voxels, MNI: *x*=10, *y*=34, *z* = 60, *Z*max = 3.68, *p*<0.001); bilateral precuneous cortex (BA7), extending in pre-central gyrus (BA5; cluster 2: 313 voxels, MNI: *x*=2, *y*=−46, *z*=66, *Z*max = 3.68, *p*=0.007); left precentral gyrus (BA6) towards middle frontal gyrus (cluster 3: 290 voxels, MNI: *x*=−28, *y*=−10, *z*=58, *Z*max = 3.37, *p*=0.012); and bilateral superior frontal gyrus (BA6; cluster 4: 257 voxels, MNI: *x*=−24, *y*=−8, *z*=76, *Z*max = 3.09, *p*=0.025).

##### Group × task interaction (*Z* > 2.3, *p* < 0.05 corrected; reappraise vs. maintain; lithium vs. placebo; Fig. 3)

There was significantly greater coupling between the right MTG (red) and the left middle frontal gyrus (covering BA 8, 6, and 9; orange) in the lithium group compared to placebo during reappraisal versus maintain (cluster 264 voxels, *Z*max = 3.46, MNI *x* = −36, *y* = 20, *z* = 46, *p* = 0.0213). Additionally, there was a greater coupling between the right vlPFC (blue) and a cluster expanding from the right caudate into the right frontal pole/anterior PFC (including voxels belonging to BA9, BA10, BA46, BA45) and ACC (BA32) frontal opercular cortex/insula (BA13; orange) for those in the placebo group compared to lithium during reappraisal compared to maintain (cluster 1130 voxels, MNI: *x* = 14, *y* = 22, *z* = 14, *Z*max = 4.27, *p* < 0.001).

**Fig. 3:**
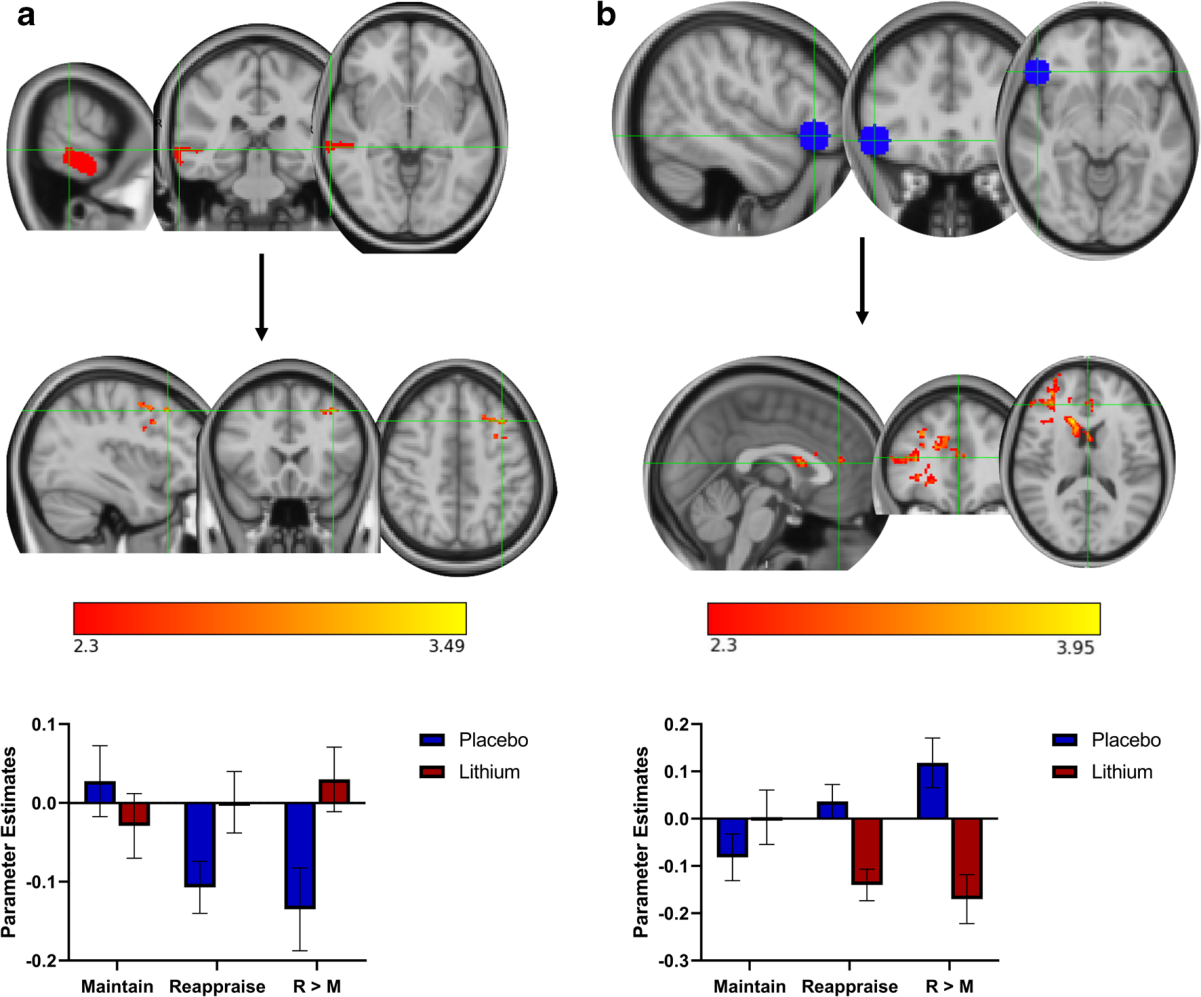
Whole-brain connectivity analysis. Group by task interaction, *Z*>2.3, *p* < 0.05 corrected. **a** There was significantly greater connectivity between the right MTG (red) and the left middle frontal gyrus (covering BA 8, 6, and 9; orange) in the lithium group compared to placebo during reappraisal versus maintain. **b** There was a significantly greater connectivity between the right vlPFC (blue) and the right caudate, extending into the right frontal pole/anterior PFC and ACC frontal opercular cortex/insula (BA13; orange) in the placebo group compared to the lithium group when reappraising versus maintain

##### Main effect of group (*Z*> 2.3, *p* < 0.05 corrected, placebo > lithium, during picture blocks (M+R>B); Fig. 4)

Greater negative connectivity/anticorrelation was found between the left amygdala and bilateral frontal cortex for the lithium group compared to the placebo group in response to aversive pictures (cluster 1 (left): 834 voxels, MNI *x*=−18, *y*=30, *z*=24, *Z*=4.37, *p*<0.001; cluster 2 (right): 519 voxels, MNI *x*=20, *y*=38, *z*=0, *Z*=4.13, *p*<0.001). Additionally, the lithium group showed greater connectivity between right MTG and bilateral PFC extending towards paracingulate gyrus than the placebo group (cluster: 326 voxels, *Z*max = 3.58, MNI *x*=−2, *y*=56, *z*=10, *p*=0.006).

**Fig. 4:**
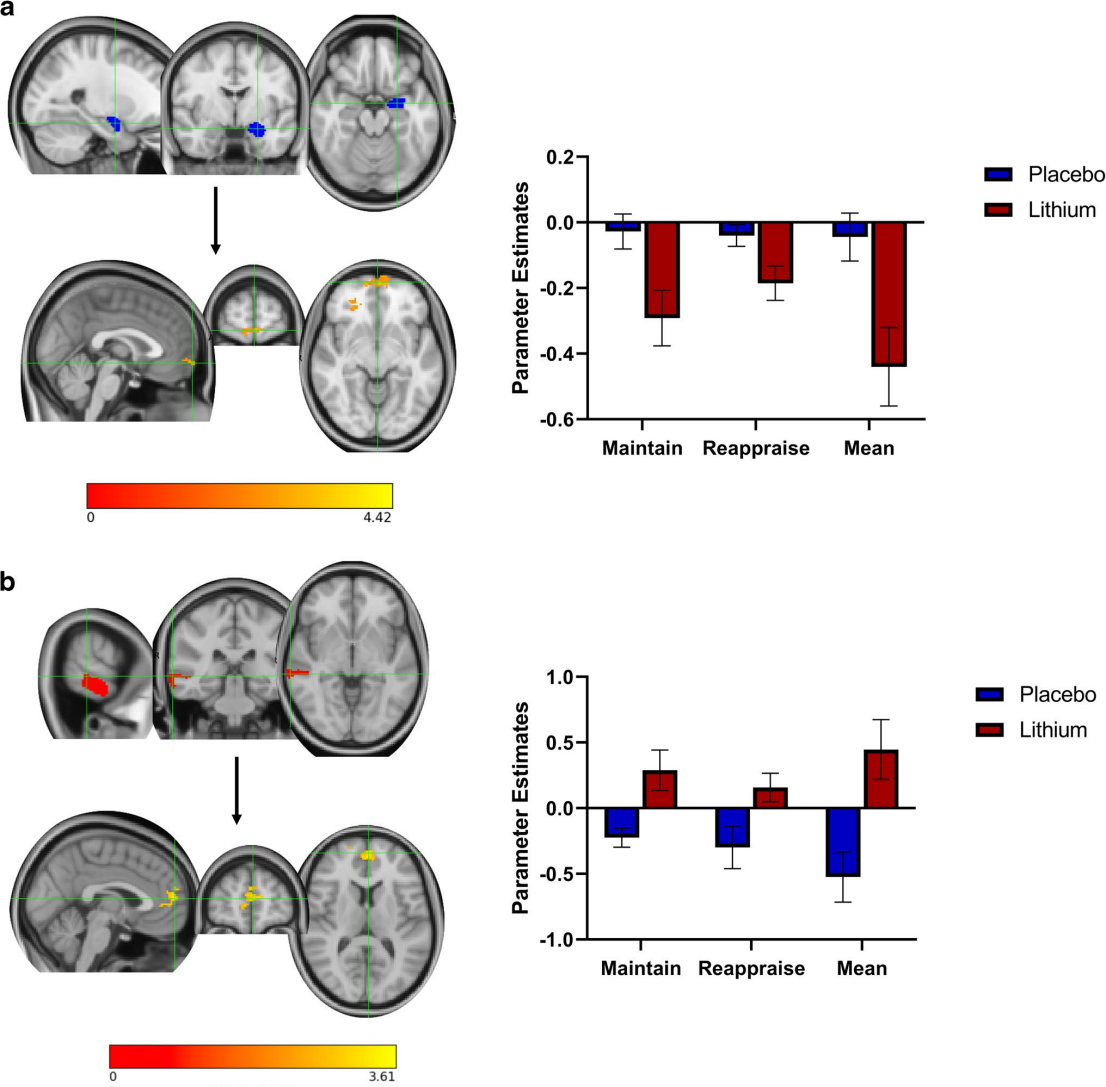
Whole-brain connectivity analysis. Main effect of group (*Z* > 2.3, *p* < 0.05 corrected). **a** There was significantly greater negative connectivity between the left amygdala (blue) and bilateral frontal cortex (orange) for the lithium group compared to the placebo group in response to aversive pictures (mean of reappraise and maintain). **b** There was significantly greater connectivity between the right MTG (red) and the bilateral medial PFC (yellow) during overall negative picture blocks (mean reappraise and maintain) in the lithium group, compared to placebo. Error bars represent SEM

#### Control analyses

##### Grey matter

Results for the task fMRI analysis remained similar when GM maps were not added into the GLM as a nuisance regressor.

##### Checkerboard control task

As reported in Volman et al. (2021), across groups, visual stimulation was associated with a large and highly significant activation cluster in the occipital cortex, among others. However, there were no significant group differences in brain activity during visual stimulation, suggesting that the observed effects during the ER task did not reflect haemodynamic changes due to lithium treatment.

## Discussion

This study evaluated the effects of lithium on ER in healthy participants. Neural effects of reappraisal, compared to naturally experiencing the negative stimuli, were seen, as expected, in frontal, temporal, parietal, and occipital regions, alongside lower self-reported negative affect ratings. Lithium’s effect on active reappraisal and emotional processing of negative images was seen in areas of the fronto-parietal and limbic network, and in superior and medial temporal structures. Within the fronto-parietal network, during reappraisal compared to maintain, lithium decreased activation in prefrontal areas (left anterior PFC or rostra-lateral PFC, and right superior frontal gyrus) and posterior parietal areas (left AG). In addition, exploratory PPI analyses revealed connectivity changes within prefrontal and limbic areas following lithium administration. These results show that lithium can affect the neural underpinnings of emotional regulation, though not in line with our a priori hypothesis, which may be useful for future neuropsychological models of mood stabilising action.

### Lithium-derived effects on ER

During reappraisal of negative images, compared to maintain, lithium exerted a number of effects on the fronto-parietal network: (1) decreased activation in the left anterior prefrontal cortex, right superior frontal gyrus, and the left AG, and (2) decreased the connectivity between the right vlPFC and other regions of the fronto-limbic cortex, including the anterior PFC, ACC, caudate, and insula. The anterior prefrontal cortex is a high-level integrative area receiving information from somatosensory networks (Peng et al. 2018) and connecting the limbic system with medial prefrontal regions (Gilbert et al. 2006; Mitchell 2011). Contributing to sensory and emotion detection, emotional processing, and regulation strategies (Gilbert et al. 2006; Mitchell 2011; Peng et al. 2018), this region is key in the top-down modulation of the emotional response (Phan et al. 2002; Mitchell 2011). Other areas of the PFC, such as the vlPFC, are also involved in ER supporting the selection or inhibition of appropriate responses (Buhle et al. 2014). As with the anterior PFC, effective functional connectivity between the vlPFC and other regions of the fronto-limbic network during ER has been reported (Townsend and Altshuler 2012). Prefrontal regions of the cortex send direct excitatory inputs to the striatum (i.e. caudate nucleus) and other regions of the basal ganglia. These areas ultimately support limbic function by contributing to the assessment of affective valence and the formation of an emotional state (Pierce and Péron 2020). The right superior frontal gyrus has previously shown to be active during tasks involving introspection or self-reflection, where participants were asked to analyse their feelings after being exposed to emotional stimuli (Briggs et al. 2020). The AG is also regarded as a cross-modal integrative hub, and has been linked to multiple functions (Seghier 2013). Previous research in the context of ER has elucidated its implication in cognitive reappraisal through the allocation of attentional resources, monitoring the emotional experience (Picó-Pérez et al. 2017), and imagination of scenes that might facilitate ER (Kohn et al. 2014).

BD has been associated with reduced recruitment of the regulatory fronto-parietal network during cognitive reappraisal (Zhang et al. 2018a) and we therefore hypothesised that lithium would have the opposing effect (i.e. increase the fronto-parietal network’s modulatory efforts). However, the observed pattern of decreased anterior PFC and AG response as well as decreased connectivity between the vlPFC and other regions of the fronto-limbic network during reappraisal following lithium administration was opposite to this hypothesis. Further work is needed to unpack this effect in more detail. For example, the effects of lithium may be modulated by the pre-existing level of ER. The healthy volunteers in our study tended to use effective ER strategies prior to treatment and lithium may have led to a disturbance in this process (i.e. a shift to the right in an inverted U-shaped function). Alternatively, reduced activation in relevant neural networks, whilst maintaining similar performance levels in cognitive tasks, has been associated with beneficial effects of treatment in other settings, presumably reflecting increased ease or efficiency of neural networks supporting cognitive function (Miskowiak and Petersen 2019). As such, the observed effects of lithium may reflect reduced need for PFC-mediated regulation to reappraise the negative pictures included here, or a reduction in the difference in regulation strategy employed between the reappraise and maintain conditions. However, this can only be resolved by assessing the effects on a behavioural task with sufficient sensitivity to detect any drug effect and/or by exploring the effects of lithium in patients with BD or a group with impaired ER at baseline.

During negative picture block presentation (collapsing across conditions), further fronto-limbic effects were seen. That is, participants in the lithium group, compared to those in the placebo group, had a significantly greater anticorrelation between the left amygdala and bilateral prefrontal regions. This anticorrelated pattern between prefrontal and limbic regions is expected when successfully employing ER strategies (Kanske et al. 2011; Paschke et al. 2016; Sarkheil et al. 2019). In patients with BD, a weaker modulatory effect of prefrontal regions, compared to healthy controls is seen (Zhang et al. 2018a), with antidepressant treatment (chronotherapy) increasing these regions’ functional connectivity in this population (Vai et al. 2015). This increase in functional connectivity has been proposed by the authors to be a potential biomarker for treatment efficacy in BD. The lithium-derived anti-correlation between the amygdala and bilateral PFC regions triggered by negative pictures without explicit regulatory instructions is consistent with the idea above that lithium may enhance regulation across conditions, irrespective of these explicit instructions, though the direction of this effect is impossible to confirm with the PPI analysis used here.

Altogether, the described effects of lithium on fronto-parietal activity and connectivity suggest that, when instructed to engage in ER strategies, prefrontal activation is reduced, potentially reflecting a lower need of regulatory efforts over limbic regions due to lithium treatment or reduced difference between the regulatory processes applied across different conditions. Consistent with this, there was a greater anti-correlation between the amygdala and PFC during viewing of negative pictures whether this was during the maintain or reappraise condition.

Further effects of the mood stabiliser on reappraisal and emotional processing of negative images can be seen on superior temporal structures. Accordingly, during reappraisal, as compared to maintain, those in the lithium group showed increased activation in the right superior temporal gyrus, and a significantly greater connectivity between the MTG and the left middle frontal gyrus. During negative picture blocks, those in the lithium group had greater correlated activation between the right MTG and bilateral medial PFC, including the paracingulate cortex and the dmPFC. Involvement of the superior and the middle temporal gyrus in emotional downregulation through cognitive reappraisal has previously been reported (Kohn et al. 2014; Buhle et al. 2014; Picó-Pérez et al. 2017; Nguyen et al. 2019). Cognitive control regions engaging medial and superior temporal structures to alter the semantic meaning and perceptual representation of negative stimuli is thought to mediate emotion downregulation (Buhle et al. 2014). In line with this, posterior areas of the prefrontal cortex, including middle frontal gyrus, are implicated in ER by directing attention to reappraisal-relevant stimulus features and holding in mind reappraisal goals, as well as the content of one’s appraisal (Ochsner et al. 2012). Both dmPFC and paracingulate areas of the prefrontal cortex have been involved in emotion processing, with the former involved in appraising other’s mental states and traits, and the latter appraising viscero-sensory signals related to subjective emotional feelings (Dixon et al. 2017). Possibly, connectivity between MTG and prefrontal regions could reflect the former making use of information pertaining to the individual and other’s (stimulus related) emotional states to effectively process and/or alter the stimuli’s meaning. Additionally, activation in MTG has been reported to affect amygdala activity (Kanske et al. 2011; Kohn et al. 2014). Lithium’s effect on the right superior and middle temporal gyrus, and its connectivity with cognitive control regions is consistent with alterations in the emotional significance of the presented stimuli, and the reduced activation of the prefrontal network described above.

### Task-related findings and general implication for emotional regulation

Increased activation of prefrontal regions, including bilateral dlPFC, vlPFC, lateral OFC, ACC, and supplementary motor area and temporal, parietal, and occipital regions, as well as subcortical structures, was observed during reappraisal of negative stimuli across groups. This task activation is consistent with previous literature investigating ER through reappraisal (Ochsner et al. 2002; Phan et al. 2005; Frank et al. 2014; Buhle et al. 2014; Reinecke et al. 2015; Paschke et al. 2016; Morawetz et al. 2017; Picó-Pérez et al. 2017; Nguyen et al. 2019; Hassa et al. 2021). Behaviourally, reappraisal successfully decreased self-reported negative affect experienced throughout the task, which adds to the existing body of literature on cognitive reappraisal as an effective strategy to downregulate the experience of negative affect (Paschke et al. 2016; Ma et al. 2017; Cao et al. 2020; Yang et al. 2020; Anand et al. 2020).

However, and contrary to previous studies (Ochsner et al. 2002; Phan et al. 2005; Kanske et al. 2011; Frank et al. 2014; Buhle et al. 2014; Picó-Pérez et al. 2017), no decreased activation of limbic structures was observed during reappraisal compared to maintain across groups. SVC analysis of the bilateral amygdala showed increased activation during reappraisal compared to maintain, across groups and hemispheres. With the use of the same task (Reinecke et al. 2015), or similar (Sarkheil et al. 2019; Hassa et al. 2021), Reinecke et al. (2015) did not report amygdala deactivation during reappraisal of negative stimuli across their sample, with Sarkeil (2019) and Hassa et al. (2021) reporting an increase. Post hoc analysis implemented by Sarkeil (2019) investigated this unexpected effect by dividing the reappraisal period into early and late to observe when the activation was occurring. Results showed that during late periods of reappraisal, amygdala activation was most increased and amygdala-prefrontal connectivity most reduced. Similar to the present study, numerous images were presented consecutively in each block, with reappraisal needing to be implemented with each individual image. The author proposed that this could have increased the cognitive load during the reappraisal block, leading to poor efficacy of frontal regions downregulating amygdala’s activation, ultimately heightening its activation during reappraisal. Given the similarity between the task used in the present study and in Sarkeil’s (2019), this could explain amygdala’s activity during reappraisal. Alternatively, Wager and colleagues’ meta-analysis (2003) found a left lateralization of the amygdala for processing negative emotions. Given the negative nature of the stimuli, it is proposed that prefrontal efforts to downregulate amygdala activity might have been lateralised to the left amygdala, leading to a higher right amygdala activation during reappraisal. Further evidence showed the right amygdala to be negatively correlated with areas of the occipital cortex and posterior parietal lobe as well as right precentral gyrus towards frontal gyrus (BA6 + 9) during reappraisal across groups. Taken within the hypervigilance model (Hofmann et al. 2012), which proposes that hyperactivation of the amygdala towards threat facilitates visual processing in the occipital cortex, reappraisal of negative stimuli could have decreased the connectivity between the amygdala and occipital regions in an effort to discontinue the feed of visual information to the amygdala. Additionally, a negative connectivity between the right amygdala and right precentral gyrus towards frontal gyrus (BA6 + 9) might reflect the latter motor planning areas (Briggs et al. 2020) planning to remove oneself from the fearful situation, thus decreasing amygdala’s activation. However, reasons behind the lateralisation of this effect to the right amygdala are not clearly understood.

Consistent with previous literature (Ochsner et al. 2012; Buhle et al. 2014), and contrary to others (Diekhof et al. 2011), during reappraisal of negative stimuli, the vmPFC (bilaterally) was not found to be active neither at whole-brain nor at SVC. This was opposite to the MTG, which was shown to be active in both analyses. Additionally, during reappraisal, the vmPFC was bilaterally correlated with a deactivation of the left vmPFC towards rostral mPFC. This possibly indicates a null involvement of this region during reappraisal. It has been previously conjectured that given the vmPFC involvement during both maintain and reappraisal, the nature of the contrast (reappraise vs. maintain) might not be specific enough to show its involvement during reappraisal (Buhle et al. 2014). The correlation of the bilateral vmPFC ROI with deactivation in regions mainly including the left vmPFC, which, however, might indicate that its involvement in reappraisal is potentially absent, or that given its role in self-related emotion generation (Dixon et al. 2017); it might not be involved in reappraisal due to the non-self-related nature of the negative stimuli of the present task. Altogether, this evidence contradicts previous hypothesis assigning the vmPFC a role as the key link between frontal and parietal regions in emotion downregulation (Diekhof et al. 2011), and further supports the hypothesis that reappraisal engages temporal structures (namely MTG) to alter the meaning of the perceived stimuli and therefore downregulate negative emotions (Buhle et al. 2014).

### Study’s strengths, limitations, and future directions

Notably, and to the best of the researchers’ knowledge, this study is the first to evaluate the effects of lithium on ER in healthy participants. This is considered to aid the understanding of the mechanisms behind lithium treatment due to the lack of interactions between treatment and disorder-related factors (i.e. disorder severity, duration, state, and/or history of medication).

The lack of treatment-derived differential effects in self-reported negative affect, however, limits the establishment of whether lithium’s neural effects were beneficial or detrimental to the overall ER process. Importantly, the absence of treatment-derived self-reported effects does not necessarily imply that differences do not exist, but perhaps that the measurement was not sensitive enough to detect them. Establishing the directionality of these effects is of importance to further understand lithium’s mood-stabilising properties. Another important limitation when interpreting the connectivity results is that the PPI analysis employed does not specify whether the seed region is the driver or the driven area, or if the connection is direct or mediated by other areas. Therefore, interpreting the causality or directionality of these results is not possible and they should be considered with caution. Respective to the study sample, and although a healthy volunteer sample poses some benefits mentioned above, these individuals may have an already near-optimal ability to regulate their emotions and so the effects of lithium may not accurately represent what would be seen with treatment in BD. Additionally, we had a relatively small sample size (*n*=33) which may have impacted the statistical power of the study for both type 1 and 2 errors. Despite being able to compare the study findings with previous literature, the lenient statistical significance threshold of *Z* > 2.3 could have resulted in false positive findings. These findings therefore need to be replicated in larger sample sizes to confirm. Although the use of the CCT task allows to control for treatment-related confounders on brain activation, the lack of a neutral condition in the ER task does not allow the control of said confounders on higher order visual processing of complex scenes. The data reported in this study was collected in 2011, and so the study and its hypotheses were not pre-registered. Lastly, we used a short treatment administration period, which may have hindered lithium’s potential to impact ER in healthy participants.

Considering the study’s limitations and the potential implications of these results for research in BD treatment, future work should validate the present evidence in a larger sample size, with a longer treatment administration, and including the assessment of physiological measurements (i.e. heart rate and/or arousal) that could inform if the implementation of reappraisal in any group is more successful. Moreover, prospective work should consider the inclusion of a condition involving the downregulation of positive stimuli. Altogether, these future recommendations could broaden the understanding of how lithium achieves mood-stabilisation in BD through ER. Lastly, and of general clinical implication, is the AG’s role in ER. Given lithium’s downregulating effect in the left AG during reappraisal and considering similar downregulation patterns observed in clinical populations (Picó-Pérez et al. 2017), further attention should be drawn to the AG’s role in ER and how its downregulation might be related to symptomatology in different mood, including BD, and anxiety disorders.

## Conclusions

The present research examined the neural effects of an 11-day lithium administration on ER in healthy participants. Lithium-derived differences in brain activation and connectivity during reappraisal (vs. maintain) were (1) decreased activation in the left AG, left anterior prefrontal cortex and right superior frontal gyrus, and connectivity between the vlFPC and caudate and other prefrontal areas; (2) increased activation in the right superior temporal gyrus (SVC); and correlated activation between the right MTG and the left middle frontal gyrus. During negative picture blocks, lithium-derived differences in brain activity were (1) increased amygdala-prefrontal anticorrelation, and (2) enhanced MTG-prefrontal connectivity. These results provide preliminary support for the involvement of lithium in emotional processing and regulation in healthy participants, and potentially benefit future work in the development of more effective treatments for BD.

Of general implications for ER and, specifically for cognitive reappraisal, the present research replicated previous findings. Consistently, enhanced activation in prefrontal including cognitive control regions, temporal, parietal, and occipital regions was found. Contrary to leading evidence, but in line with a small number of studies, no decreased in amygdala’s activity was found, with the right amygdala observing an increase during reappraisal, compared to maintain. Lastly, the present research shows support for Buhle et al.’ (2014) hypothesis implicating temporal structures, as opposed to the vmPFC, in the downregulation of negative stimuli through reappraisal.

Taken together, these results show an effect of lithium in emotional processing and regulation, and further elaborate the neural underpinnings of cognitive reappraisal. Although limitations should be considering when interpreting these results, a call for future research to investigate longer term effects of lithium on ER in individuals with BD is made, which ultimately may benefit the development of novel and more effective treatments for BD.

## Supplementary information

ESM 1 (DOCX 345 kb)

## Declarations

### Disclaimer

The views expressed here are those of the authors and not necessarily those of the NHS, the NIHR or the Department of Health.
