# Supplementary material for: Effect of lithium administration on brain activity under an emotion regulation paradigm in healthy participants: a functional magnetic resonance imaging study
Source: Psychopharmacology (Berl). 2023 Jun 20;240(8):1719–34. doi: 10.1007/s00213-023-06395-7 (PMC10349753; doi:10.1007/s00213-023-06395-7)
Supplement: Supplementary file 1 — ESM 1 (DOCX 345 kb) [file 213_2023_6395_MOESM1_ESM.docx]

**Effect of Lithium Administration on Brain Activity Under an Emotion Regulation Paradigm in Healthy Participants: A Functional Magnetic Resonance Imaging Study**

**Supplementary material**

**Methods:**

**Participants**

In addition to the exclusion criteria presented in the methods section of the paper, participants were excluded from taking part in the study if they had any medical contraindication. This was for example conditions that might alter absorption of lithium or which could impact on the safety of the drug for the volunteer, such as impaired renal function as assessed by creatinine levels or impaired thyroid function as assessed by thyroid stimulating hormone levels. In addition participants were excluded if they took any medication that could cause clinically significant interactions with lithium like: ACE inhibitors, NSAIDs, angiotensin-II receptor antagonists, sodium bicarbonate, amiodarone, metronidizole, SSRI, carbamazepine, phenytoin, antipsychotic drugs, diuretics, methyldopa, muscle relaxants, baclofen, neostigmine, pyridostigmine and theophylline).

**Tasks**

**Emotion Regulation Task (Reinecke et al., 2015)**

40 negatively valanced coloured International Affective Picture System (IAPS; Lang et al., 1997) images portraying characteristic panic- related catastrophic expectations, such as accidents or attacks (mean valence rating of 2.8±1.7, mean arousal ratings of 6.0±2.2 on 9-point Likert scales from 1=unpleasant/low arousal to 9=pleasant/high arousal) were used. Images were presented in eight blocks of five images, each displayed for five seconds and separated by one-second fixation cross. Image blocks were alternated with a baseline block (fixation cross) of thirty seconds, where participants were asked to relax. Starting with a baseline block, there were two alternating experimental conditions: maintain (M), where participants were instructed to passively view the images and naturally experience the emotional state evoked; and reappraise (R), where participants were instructed to downregulate the provoked negative affect through reappraisal, trained prior to the scanning session. Half of the participants per group (placebo/lithium) started with maintain and half with reappraise. Instructions were given before the block by presenting the word “maintain” or “suppress” for four seconds. Following each picture block, a 4-point rating scale (1=neutral; 4=negative) was displayed for four seconds and participants had to indicate using a keypad the intensity of the negative affect experienced throughout the block (see Figure 1 for an example trial).

**Checkerboard Control Task (Murphy et al., 2009)**

The task consisted of two conditions: flash and baseline, with 10 blocks of alternating black and white squares switching at a frequency of eight Hz; and 10 blocks of a fixed-cross, respectively. Each flash block lasted for 16 seconds and each baseline block of 15 seconds. Participants were instructed to lay still while keeping their eyes open and blinking normally.

**Figure S1.**

*Emotion Regulation Task: Example Trial.*


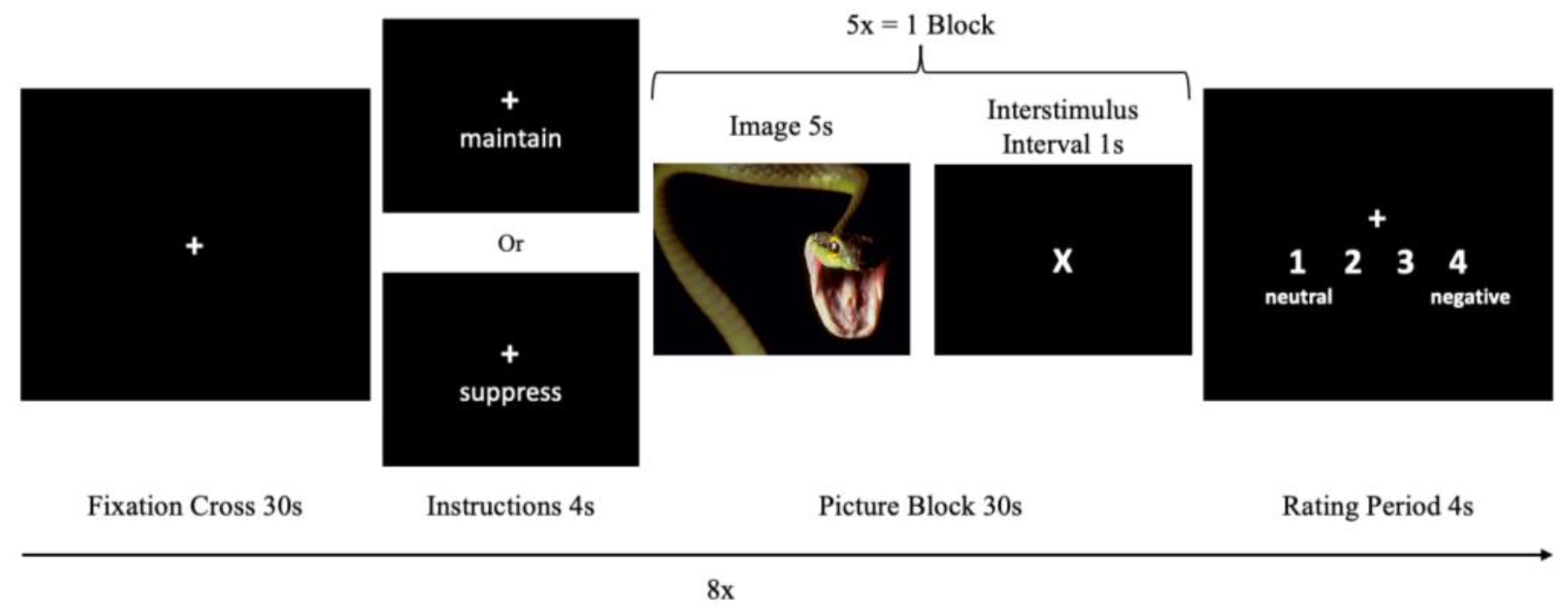


*Note*: the task starts with fixation cross for 30 seconds followed by instructions indicating either suppress or maintain. Picture Block consists of 5 images, 5 seconds each, each separated by 1s interstimulus interval. At block completion, a rating period of 4 seconds takes place.

**Results**

Table S1 – Main effect of the emotion regulation task across groups

|  | **Brain area** | **Side** | **Cluster size (voxels)** | **MNI max (x, y, z)** | ***z-*score** | ***p*-value** |
| --- | --- | --- | --- | --- | --- | --- |
| **Task activation (mean across groups, reappraise > maintain) (***Z* > 3.1, *p* < 0.05) |  | | | | | |
| Cluster 1 | Paracingulate cortex  ACC, supplemetary motor cortex and dmPFC.  dlPFC, vlPFC and lateral OFC. Insular cortex and frontal operculum, hippocampus, thalamus, cerebellum, lateral occipital cortex and the superior and middle temporal gyrus, supramarginal gyrus and angular gyrus | R+L | 36268 | 14, 20, 58 | 6.88 | <0.001 |
| Cluster 2 | Occipital pole | R+L | 268 | 4, -94, 4 | 4.26 | <0.001 |
| Cluster 3 | Cingulate gyrus | L | 140 | -4, -26, 28 | 4.81 | 0.0015 |
| Cluster 4 | Cingulate gyrus, precuneous cortex, intracalcarine cortex | R | 110 | 0, -42,18 | 3.96 | 0.0068 |
| Cluster 5 | Inferior temporal gyrus, temporal pole | R | 102 | 50, 2, 42 | 3.94 | 0.0104 |
| **Task activation (mean across groups, reappraise > maintain) (***Z* > 2.3, *p* < 0.05) | | | | | | |
| Cluster 1 | In addition to regions above: anterior PFC (BA10), posterior CC, amygdala, insular cortex | R+L | 85337 | 50, 2, -42 | 7.13 | <0.001 |
|  | MNI coordinates (*x, y, z*) refer to the peak of activation within each cluster | | | | | |

Table S2 – Small Volume Correction Significant Increased Activation in Selected Regions of Interest. Z>3.1, p<0.05 Corrected

|  | **Brain area** | **Side** | **Cluster size (voxels)** | **MNI max (x, y, z)** | ***z-*score** | ***p*-value** |
| --- | --- | --- | --- | --- | --- | --- |
| *Z* > 3.1, *p* < 0.05 |  | | | | | |
| **Task activation**  **Mean across groups**  **R > M** | Amygdala | R | 11 | 26, -6, -18 | 3.79 | 0.0074 |
|  | Amygdala | R | 7 | 22, 0, -26 | 3.72 | 0.0118 |
|  | Amygdala | R | 1 | 26, 0, -20 | 3.17 | 0.0324 |
|  | Amygdala | L | 25 | -26, -6, -16 | 3.80 | 0.0016 |
|  | vlPFC | R | 287 | 52, 24, -2 | 6.24 | <0.001 |
|  | vlPFC | L | 483 | -44, 18, -4 | 6.38 | <0.001 |
|  | MTG | R | 167 | 52, -36, -4 | 4.86 | <0.001 |
|  | MTG | L | 344 | -50, -34, 4 | 4.97 | <0.001 |
| **Group x Task**  **(R > M; L > P)** | MTG extending into STG | R | 18 | 52, -32, 4 | 4.52 | 0.0064 |
| **Aversive pictures (mean R+M > baseline)**  **P > L** | vlPFC | R | 2 | 52, 34, -2 | 3.48 | 0.0413 |
|  |  | R | 1 | 54, 26, -2 | 3.49 | 0.050 |
|  | vlPFC | L | 2 | -48, 28, -4 | 3.32 | 0.0414 |
|  |  | L | 2 | -42, 28, 0 | 4.77 | 0.0414 |
|  |  |  |  |  |  |  |
| *Note*: MTG: middle temporal gyrus, STG: superior temporal gyrus; R > M, reappraisal versus maintain; ROI, region of interest; MNI, Montreal Neurological Institute; R, right; L, left. MNI coordinates (*x, y, z*) refer to the peak of activation within each cluster | | | | | | |

**Checkerboard Control Task Results.**

In the CCT across groups, visual stimulation was associated with a large and highly significant activation cluster in the occipital cortex (cluster: 25516 voxels, MNI x=8, y=-86, z=-10, *Z*=9.58, *p*<0.01) in addition to regions including the bilateral primary motor area, bilateral supplementary motor area, bilateral cerebellum, bilateral thalamus, bilateral putamen, bilateral frontal operculum and right supramarginal gyrus (for full details see Table S3)

Table S3 – Main effect of the checkerboard control task across groups

|  | **Brain area** | **Side** | **Cluster size (voxels)** | **MNI max (x, y, z)** | ***z-*score** | ***p*-value** |
| --- | --- | --- | --- | --- | --- | --- |
| **Task activation mean across groups, flashes > baseline** *Z* > 3.1, *p* < 0.05 |  | | | | | |
| Cluster 1 | Occipital cortex, supramarginal gyrus, | R+L | 25516 | 8, -86, 10 | 9.58 | <0.001 |
| Cluster 2 | Putamen, frontal operculum cortex, precentral gyrus, frontal pole, middle frontal gyrus, inferior frontal gyrus | R | 3449 | 22, 12. 8 | 5.78 | <0.001 |
| Cluster 3 | Cerebellum | L | 905 | -28, -66, -44 | 6.00 | <0.001 |
| Cluster 4 | Precentral gyrus, middle frontal gyrus, inferior frontal gyrus | L | 530 | -46, 4, 38 | 5.37 | <0.001 |
| Cluster 5 | Thalamus | R | 441 | 22, -28, 0 | 7.64 | <0.001 |
| Cluster 6 | Middle frontal gyrus, superior frontal gyrus, | L | 397 | -32, -2, 66 | 4.79 | <0.001 |
| Cluster 7 | Frontal operculum cortex, extending into insular cortex and left putamen | L | 383 | -36, 24, 4 | 5.61 | <0.001 |
| Cluster 8 | Cerebellum | R | 347 | 26, -66, -46 | 4.74 | <0.001 |
| Cluster 9 | Superior frontal gyrus | R | 329 | 12, 4, 68 | 4.53 | <0.001 |
| Cluster 10 | Thalamus | L | 322 | -22, -26, -6 | 7.48 | <0.001 |
| Cluster 11 | Supramarginal gyrus | R | 98 | 48, -40, 18 | 3.82 | 0.022 |
| MNI coordinates (*x, y, z*) refer to the peak of activation within each cluster | | | | | | |
